# A study on nonlinear estimation of submaximal effort tolerance based on the generalized MET concept and the 6MWT in pulmonary rehabilitation

**DOI:** 10.1371/journal.pone.0191875

**Published:** 2018-02-09

**Authors:** Jan Szczegielniak, Krzysztof J. Latawiec, Jacek Łuniewski, Rafał Stanisławski, Katarzyna Bogacz, Marcin Krajczy, Marek Rydel

**Affiliations:** 1 Department of Physical Education and Physiotherapy, Opole University of Technology, Opole, Poland; 2 Department of Electrical, Control and Computer Engineering, Opole University of Technology, Opole, Poland; Bloom Technologies, BELGIUM

## Abstract

**Background:**

The six-minute walk test (6MWT) is considered to be a simple and inexpensive tool for the assessment of functional tolerance of submaximal effort. The aim of this work was 1) to background the nonlinear nature of the energy expenditure process due to physical activity, 2) to compare the results/scores of the submaximal treadmill exercise test and those of 6MWT in pulmonary patients and 3) to develop nonlinear mathematical models relating the two.

**Methods:**

The study group included patients with the COPD. All patients were subjected to a submaximal exercise test and a 6MWT. To develop an optimal mathematical solution and compare the results of the exercise test and the 6MWT, the least squares and genetic algorithms were employed to estimate parameters of polynomial expansion and piecewise linear models.

**Results:**

Mathematical analysis enabled to construct nonlinear models for estimating the MET result of submaximal exercise test based on average walk velocity (or distance) in the 6MWT.

**Conclusions:**

Submaximal effort tolerance in COPD patients can be effectively estimated from new, rehabilitation-oriented, nonlinear models based on the generalized MET concept and the 6MWT.

## Introduction

Even though the double labeled water method is considered the most general technique to estimate energy expenditure due to physical activity [1], it is commonly accepted that using the VO2max is a valid measure of functional capacity for patients with cardiopulmonary diseases. Many studies have been conducted to estimate the VO2max values based on the cycle exercise test, treadmill test, walk tests [1–4] and, on the other hand, running performance [5]. However, there is also evidence that submaximal exercise testing can be inaccurate for determining VO2max which cannot be estimated by prediction equations in patients with stable COPD [6]. Searching for possible reasons for the inaccuracies we trace back to the work of Åstrand and Rodehl [7], who assumed the linear relationship between VO2max and power output during incremental exercise. The function is linear indeed, but during low power output exercise only. At (sub)maximal exercise testing performed above the anaerobic/lactate threshold (ALT) [8–10], the oxygen uptake becomes essentially nonlinear with respect to the power output [10, 11]. It is well known that the recalled anaerobic/lactate parameter has little to do with “threshold” in fact as it may vary between individuals and also it may depend on various conditions, including environmental ones. Specifically, athletes may have very high ALTs but chronically sick patients may face very low values of ALT. In any case, nonlinearity of relationship between VO2max and power output should be accounted for when precisely modeling functional capacity of populations under (sub)maximal exercise tests.

From a clinical point of view it is essential to prescribe an “optimal” level of training loads in patients with cardiopulmonary diseases. For this purpose submaximal exercise test (ET) is usually performed. It defines the amount of effort that a patient can safely perform, and allows to define exercise heart rate limit for each patient. Exercise test result is expressed in Watts or METs. The most commonly used ET protocol for patients with cardiopulmonary dysfunction is the modified Bruce treadmill protocol, where the result is expressed in METs.

One MET is defined as 1 kcal/kg/hour and is roughly equivalent to the energy cost of sitting quietly. A MET unit is also defined as oxygen uptake in ml/kg/min with one MET equal to the oxygen cost of sitting quietly, equivalent to some 3.5 ml/kg/min [12]. However, there is also evidence in literature that MET may not be a good indicator of oxygen consumption [12, 13].

Considering that 6MWT is a simple and inexpensive tool for the assessment of functional tolerance of submaximal effort and it aims at global and integrated assessment of functioning of all systems engaged in fast walking, such as respiratory, cardio-vascular and neuromuscular systems [14, 15], the 6MWT is frequently used to determine functional capacity in patients [16, 17]. Research conducted by Solway et al [18], comparing the usefulness of various walk tests for efficiency tolerance assessment, showed that the six-minute walk test is the most useful type of walk tests, best tolerated by patients and correlating with the ability to undertake daily activity efforts [4]. The six-minute walk test is considered an indicator of the ability to undertake daily activity and may be used for elderly patients and for patients with COPD and after myocardial infarction [19–23]. Additionally, 6MWT shows moderate and high (linear) correlation (0.69 < *r* < 0.91) with maximal oxygen uptake derived from a cardiopulmonary exercise test in a well-defined group of patients [24]. The question left unanswered is whether it is possible to effectively estimate the ET results on the basis of 6MWT for wide-spectrum COPD patients.

The aim of this work is to compare results of the submaximal exercise test and results of 6MWT, expressed in METs, in patients with wide-range COPD-related dysfunctions and to develop mathematical correlations between them. The study was approved by the Committee of Ethics, Opole Chamber of Physicians (No. 139/2012).

## Methods

Study population. The study included 299 patients (140 female, 159 male) with diagnosed COPD (stages I to III) treated at the Physiotherapy Department, MSW Hospital in Glucholazy, Poland, between 2013–2016 (Table 1).

**Table 1:** Baseline characteristics of the entire study sample.

Number of COPD patients	299
Average age (±SD)	56.43 (±6.13)
Average range (years)	40–65
Average body mass (±SD)	83.88 (±9.72)
Average height (±SD)	171.20 (±10.49)

The study group included patients undergoing physiotherapy, who agreed to participate in the study. Informed written consent was obtained from all patients. Exclusion criteria included absolute contraindications to initiation of physical training, fever, infections and inflammations, diseases and injuries of musculoskeletal system impairing movement, unstable diabetes and mental illnesses. All the patients were subjected to a submaximal exercise test, without stopping pharmacological treatment. On the following day, a six-minute walk test was performed. In the submaximal exercise test, HR limit for all patients was established at the level of 70 to 80% of predicted maximal heart rate. The maximal heart rate was calculated for each patient individually, using the following equation: *HR*_*max*_ = 208 − 0.7 × *age* [25].

Effort tolerance assessment was made on the basis of the ET on the Cardioperfect treadmill. The ET was carried out using the modified Bruce protocol. Fifteen patients in the group participating in the study did not achieve their determined pulse values and exercise test termination was caused by occurring subjective or objective symptoms of effort intolerance. The six-minute walk test was carried out in the corridor, 30 meters in length, closed for common use for the duration of the test. The test protocol met the ATS guidelines for the 6MWT [23].

Our reference will be treadmill exercise tests, the common tool for estimation of submaximal effort tolerance. In order to introduce to an application of the 6MWT we firstly offer the

## Unified distance/velocity-related framework for treadmill exercise tests

Denote by *T* and *D* the results of any fix-paced treadmill exercise test in minutes (walk time) and meters (walk distance), respectively.

Then we have

$$\begin{array}{c} D=\sum_{i=1}^{S}T_{s,i}v_{i}1000/60+\delta D\;\left[m\right] \end{array}$$ (1)

where

*T*_*s*, *i*_ − duration of the *i*th stage of test, *i* = 1, …, *S* + 1 [min]
*v*_*i*_ − treadmill velocity at the *i*th stage of test, *i* = 1, …, *S* + 1 [km/h] = 1000/60 [m/min] = 50/3 [m/min]
*S* − number of stages *completed*,
*δD* − distance walked during the (*S* + 1)th stage (*uncompleted*)
  $$\begin{array}{c} \delta D=\delta T_{s,S+1}v_{S+1}\;50/3\;\left[m\right] \end{array}$$ (2)
  with *δD* < *T*_*s*,*S*+1_ *v*_*S*+1_ 50/3 [m]
*δT*_*s*,*S*+1_ − walk time at the (*S* + 1)th stage (*uncompleted*)
  $$\begin{array}{c} \delta T_{s,S+1}=T-\sum_{i=1}^{S}T_{s,i}\;\left[\text{min}\right] \end{array}$$ (3)
  with *δT*_*s*,*S*+1_ < *T*_*s*,*S*+1_ [min]

The walk time

$$\begin{array}{c} T=\sum_{i=1}^{S}T_{s,i}+\delta T_{s,S+1}\;\left[\text{min}\right] \end{array}$$ (4)

converted into the test result *M* in METs (via VO2max) can now be related with the walk distance *D* as in (1) or the average walk velocity *v* = *D*/*T* [m/min] = (3/50) *D*/*T* [km/h], giving *M* = *M*_*D*_(*D*) or *M* = *M*_*v*_(*v*), respectively (instead of *M* = *M*_*T*_(*T*)). In the 6MWT environment, featuring *v*_6*M*_ = *D*/6 [m/min] = *D*/100 [km/h], they will be substituted with *M* = *M*_6*M*_(10^−2^ *D*) or, equivalently, *M* = *M*_6*M*_(*v*).

**Example**: Modified Bruce (mBruce) protocol to be used hereinafter. For the mBruce protocol we have *T*_*s*,*i*_ = *T*_*s*_ = 3 [min], *i* = 1, …, *S*, so that

$$\begin{array}{c} D=\left(50/3\right)T_{s}\sum_{i=1}^{S}v_{i}+\delta D\;\left[m\right] \end{array}$$ (5)

with *δD* = (50/3)*δT*_*s*,*S*+1_*v*_*S*+1_ [m], *δT*_*s*,*S*+1_ = *T* − *ST*_*s*_ [min] and *v*_*i*_ [m/min] = (3/50) [km/h], *i* = 1, …, *S* + 1, as specified (for particular stages) in the mBruce protocol. Now, a table for the mBruce protocol could be supplemented with e.g. distance-related data in meters (or velocity-related data in km/h) but we refrain from recalling the supplemented table for space-saving reasons. Rather, we exemplify the calculations for one specific treadmill score, e.g. *T* = 10.5 [min], *M* = 5.95 [MET], for which we have *S* = 3, *v*_1_ = *v*_2_ = *v*_3_ = 2.7 [km/h], *v*_4_ = 4.0 [km/h], *δT*_*s*,4_ = 1.5 [min], *δD* = 100 [m], *D* = 505 [m], *v* = (3/50)*D*/*T* [km/h] = 2.886 [km/h] and, reffering to the 6MWT, *v*_6*M*_ = (*D*/100) [km/h] = 5.05 [km/h]. Comparing the values of *v* and *v*_6*M*_, the example clearly supports the well-known fact that the 6MWT is less exhausting than the treadmill test.

## 6MWT models

Applications of the simple, affordable and reproducible 6MWT have been presented in such a plethora of publications that any collection of references could by no means be considered complete. We recall a selected series of recent 6MWT-related contributions covering various medical fields [26–33], in addition to pulmonary rehabilitation highlights [15, 16, 21, 34–37]. It is striking that in the overwhelming majority of those publications the linear regression tools were employed and linearized models have been used, resulting in rather high standard deviations of the involved random variables. No wonder that many limitations have been reported in the usefulness of the otherwise simple 6MWT [38–40]. We will show how to relax some of them, in particular those related to the nonlinearity of the problem.

The first mathematical model relating the energy expenditure *M* with the average patient’s velocity *v* = *v*_6*M*_ in the context of the 6MWT was proposed by Connors and Hilling [41]

$$\begin{array}{c} M_{1}=\left(1.766v+3.5\right)/3.5\;\left[\text{MET}\right] \end{array}$$ (6)

$$\begin{array}{c} =0.5046v+1\;[\text{MET}] \end{array}$$ (7)

Note that it does not matter too much whether the velocity *v* is expressed in mph or km/h as the parameter at *v* is anyway estimated. Still, we prefer the metric system since, with the walk time *T* = 6 [min], the abscissa velocity scale in km/h will be equivalent to scaling it with distance 10^−2^ *D* in meters. (Alternatively, the abscissa velocity scale in 10^2^ km/h will correspond to that for distance *D* in meters.)

It is funny that the above linear model *M*_1_ = *f*_1_(*v*) was sometimes used in the rehabilitation practice in the form (6) rather than in the simpler form (7). Today, with a quite symbolic value of the model (6) or (7), it is worth emphasizing that the model is directly related with the definition of MET, with its resting value of unity being equivalent to oxygen uptake of 3.5 ml/kg/min. Also note that the uptake value could as well be equal to e.g. 3.4 or 3.6, without affecting the unity MET value in model (7) but possibly affecting the value(s) of parameter estimate(s). This could result in possible application of a model like (7) to e.g. overweight and obese patients where the O2 uptake of 3.5 ml/kg/min may be questioned [42]. Thus, the model (7) can be considered to cover exceptional cases also, with the resting uptakes different from 3.5 ml/kg/min. In such environments, we could talk about the generalized MET concept, with the model (7) apparently supporting the idea.

## Nonlinear models

One of the first attempts at accounting for the nonlinearity in development of a model comparing the 6MWT and MET was the study conducted by Gomberg-Maitland et al., who concluded that 6MWT-related MET can be used in clinical management of less sick patients [43]. However, they employed two-segment piecewise linear models only, in addition to the application of a less useful, ‘reverse’ model *D* = *φ*(*M*), with the resting unity MET voiding. A similar nonlinear reverse characteristic, without a model however, was considered by Beatty *et al*. [44].

In contrast, a nonlinear model-based approach to oxygen uptake estimation during the incremental shuttle walk test (ISWT) for the purpose of cardiac rehabilitation has interestingly been surveyed and extended by Buckley *at al*., see [45] and references therein. However, based on our experience in modeling and identification of complex systems, also dynamical ones [46–50], we have to raise a certain reservation to their otherwise outstanding contribution. Namely, the energy expenditure process is a very complex bio-physico-chemical system, so that approximating its nonlinear statics with a very simple, two-parameter, exponential model *y* = *b* exp(*av*), where *y* and *v* are the oxygen uptake output and walking speed input, respectively, and *a* and *b* are model parameters to be estimated, cannot provide satisfactory estimation accuracy due to underfitting. In fact, the estimate of *b* equal to some 4.5 is quite far from the typical resting oxygen uptake of some 3.5 ml/kg/min. Note that if we try to match the exponential model to the generalized MET form, with the resting unity MET incorporated no matter what is the resting oxygen uptake, this would lead to the model with *b* = *b*_0_ = 1, with only a single parameter left to be estimated. This would be a disaster in terms of the extreme underfitting. Clearly, the two-parameter exponential model *y* = *b* exp(*av*) is too simple to be effectively used in the energy expenditure modeling task.

Now, the time has come to extend the model (7) to the actual, nonlinear environment. For lucidity, we will refrain from a general, stochastic formulation of our model as this would lead to the inclusion of a sophisticated statistical machinery that might blur the core of the analysis.

### Polynomial expansion model

(A deterministic part of) the polynomial expansion model can be expressed as

$$\begin{array}{c} M_{pe}=M_{pe}\left(v\right)=1+a_{1}v+...+a_{K}v^{K}\;\left[\text{MET}\right] \end{array}$$ (8)

with *K* being usually selected in a heuristic way and the unknown parameter vector *A* = [*a*_1_, *a*_2_, …, *a*_*K*_]^*T*^ being analytically least squares (LS) estimated using a linear regression formulation. Note that for *v* = 0 we have the resting MET equal to 1.

### Piecewise linear model

The *n*-segment piecewise linear model can be presented as

$$\begin{array}{c} M_{pl}=M_{pl}\left(v\right)=\{\begin{array}{cc} a_{1}v+b_{1} & 0\leq v<V_{1}\\ a_{2}v+b_{2} & V_{1}\leq v<V_{2}\;\left[\text{MET}\right]\\ \vdots & \vdots \\ a_{n}v+b_{n} & v\geq V_{n} \end{array} \end{array}$$ (9)

where *a*_*i*_, *i* = 1, …, *n*, *b*_*j*_, *j* = 2, …, *n*, are the unknown parameters and *V*_*i*_, *i* = 1, …, *n*, are the unknown change points, compare [51, 52].

Note that, due to the linear spline property of the segments, the *b*_*j*_ parameters, *j* = 2, …, *n*, can be calculated in an analytical way

$$\begin{array}{c} b_{j}=\left(a_{j-1}-a_{j}\right)V_{j-1}+b_{j-1}\;j=2,...,n \end{array}$$ (10)

with *b*_1_ = 1 resulting in *M*_*pl*_ = 1 for *v* = 0. Now, the unknown parameter vector *C* = [*a*_1_, *a*_2_, …, *a*_*n*_, *V*_1_, …, *V*_*n*_]^*T*^ could be estimated numerically using the least squares (LS) method. Unfortunately, the estimation problem is highly nonlinear with respect to the parameters *V*_1_, …, *V*_*n*_, so that the numerical LS minimization algorithm is often stuck in a local minimum. A remedy is to use a genetic algorithm (GA) which is much likely to converge to a global minimum. Luckily, the ranges of possible changes in *V*_*i*_s can be easily “guessed” by an expert designer, so this can facilitate the GA procedure. Nonetheless, we have succeeded in the development of a combined LS/GA minimization procedure which is effectively used here.

**Remark 1**. In order to discriminate between the above specified model outputs and results/scores measured from the tests we will denote the estimated model outputs with $\hat{M}$ and the measured system outputs with *M*, possibly adding the appropriate subindexes wherever necessary.

## Results

**Fig 1:**
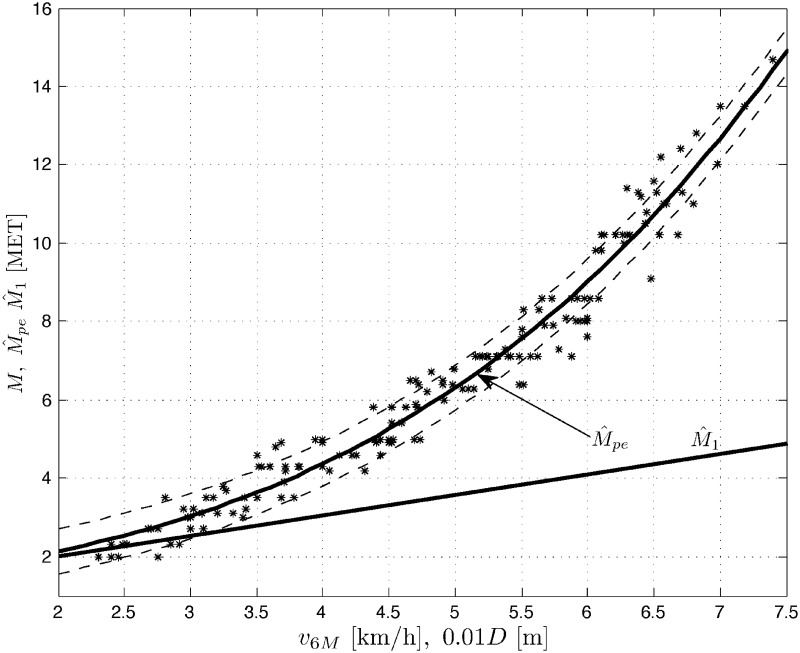
Data set *M* vs. *v*_6*M*_ (or 10^−2^ *D*) and the model outputs ${\hat{M}}_{pe}$ and ${\hat{M}}_{1}$ for men patients’ population.

**Fig 2:**
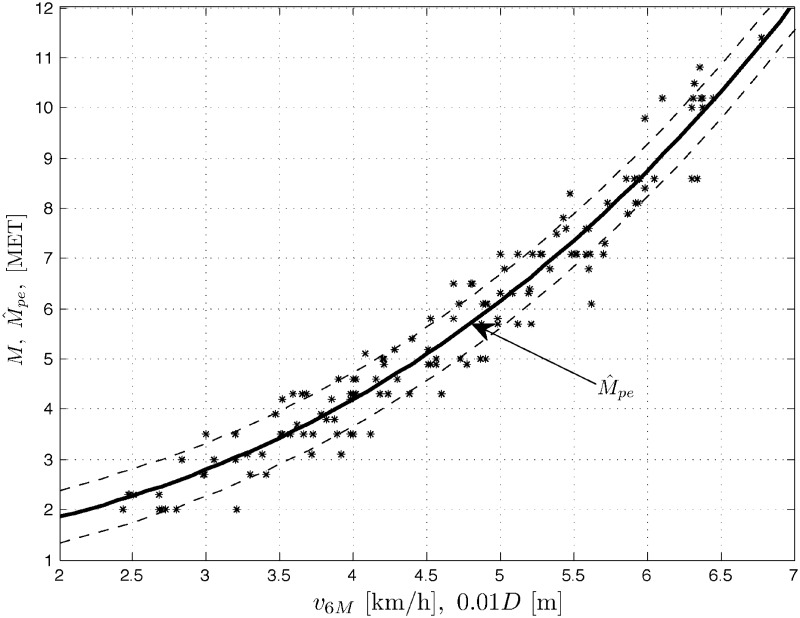
Data set *M* vs. *v*_6*M*_ (or 10^−2^ *D*) and the model output ${\hat{M}}_{pe}$ for women patients’ population.

**Fig 3:**
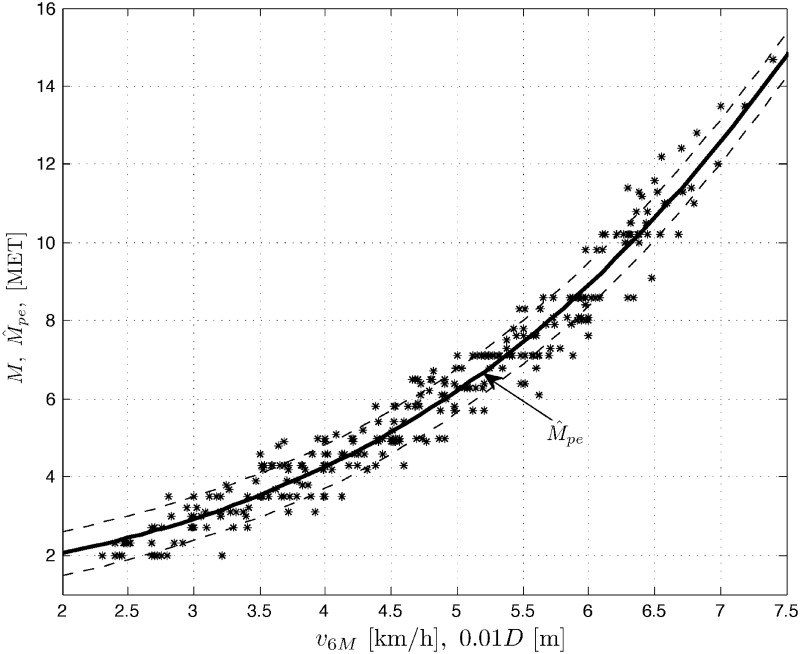
Data set *M* vs. *v*_6*M*_ (or 10^−2^ *D*) and the model output ${\hat{M}}_{pe}$ for overall patients’ population.

Results *M* of treadmill exercise tests, measured in METs vs. results of the 6MWT, expressed in velocity *v*_6*M*_ [km/h] (or distance 10^−2^ *D* [m]) are plotted for men (population *N*_*m*_ = 159), women (*N*_*w*_ = 140) and the whole COPD patient population (*N*_*p*_ = *N*_*m*_ + *N*_*w*_ = 299) in Figs 1 to 3, respectively. Three sets of data *M* vs. *v*_6*M*_ suggest nonlinear (stochastic) models. The figures also contain the corresponding outputs ${\hat{M}}_{pe}$ from the polynomial expansion models (8) for *K* = 3 and the marked standard deviation ranges (results for *K* = 5 are quite comparable). For negative comparison, the output ${\hat{M}}_{1}$ from the (very poor) linear model (7) is only once plotted in Fig 1. Below you can find the sets of the optimal LS parameter estimates $\hat{A}$ and the corresponding root mean square errors $RMSE=\sqrt{\left(1/N\right)\sum {\left[M\left(l\right)-\hat{M}\left(l\right)\right]}^{2}}$, where *l* = 1, …, *N*, is the number of consecutive samples of the result *M* and its modeled value $\hat{M}$ (whose squared difference sum is minimized by the LS method) and *N* is the total number of samples, with *N* being equal to *N*_*m*_, *N*_*w*_ or *N*_*p*_ for the populations of men, women and all the COPD patients, respectively.

$$\begin{array}{c} \begin{array}{cc} \text{Men}(N_{m}=159): & \\ \hat{A}={\left[0.51701,\;-0.029094,\;0.027643\right]}^{T}, & RMSE=0.5671\\ \text{Women}(N_{w}=140): & \\ \hat{A}={\left[0.1822,\;0.092524,\;0.015432\right]}^{T}, & RMSE=0.5288\\ \text{All}\;\text{patients}(N_{p}=299): & \\ \hat{A}={\left[0.44886,\;-0.014615,\;0.026691\right]}^{T}, & RMSE=0.5607 \end{array} \end{array}$$

It is worth mentioning that increasing the model order *K* to 9 (or higher odd) leads, unsurprisingly, to an increase in *RMSE* due to rather highly disturbed measurements *M*(*l*).

For negative comparison, the (very poor) linear model (7) applied e.g. to the men’s patient group produces *RMSE* as high as 3.3969.

**Fig 4:**
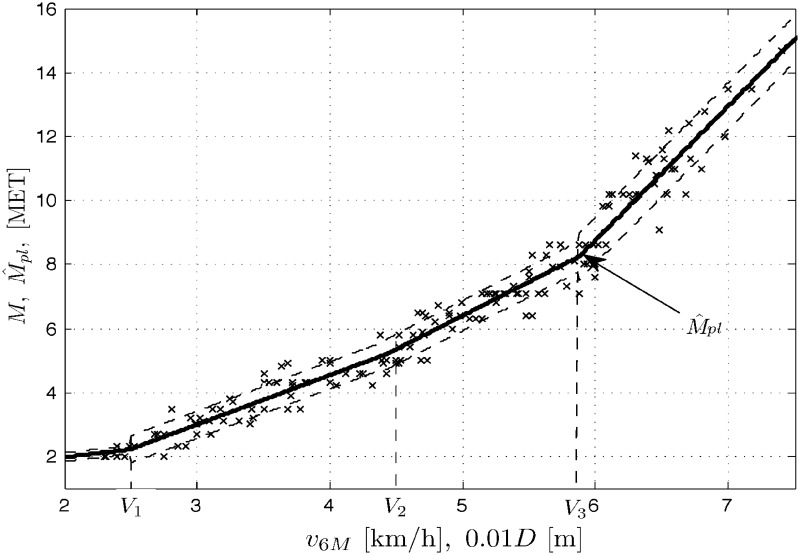
Data set *M* vs. *v*_6*M*_ (or 10^−2^ *D*) and the model outputs ${\hat{M}}_{pl}$ for men patients’ population.

**Fig 5:**
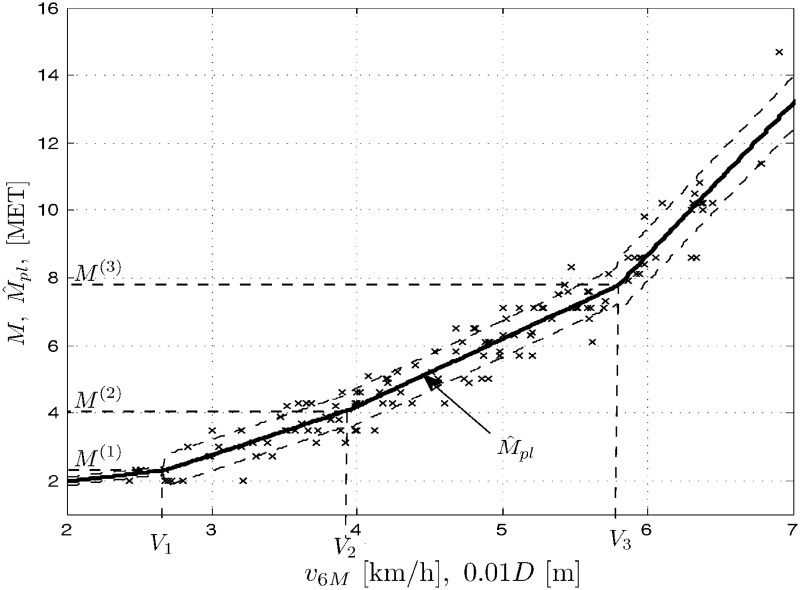
Data set *M* vs. *v*_6*M*_ (or 10^−2^ *D*) and the model outputs ${\hat{M}}_{pl}$ for women patients’ population.

**Fig 6:**
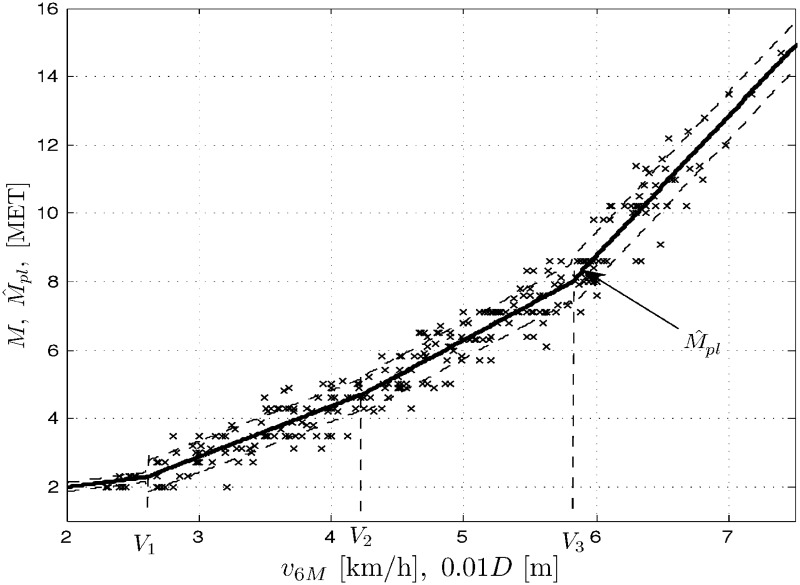
Data set *M* vs. *v*_6*M*_ (or 10^−2^ *D*) and the model outputs ${\hat{M}}_{pl}$ for overall patients’ population.

In a similar way, the modeling and simulation work was performed for the three population cases as above and the piecewise linear model (9). In addition to the data sets as above, Figs 4 to 6 present the corresponding outputs ${\hat{M}}_{pl}$ from the piecewise linear models (9) for *n* = 4 and the marked standard deviation ranges. Below you can find the sets of the optimal LS/GA parameter estimates $\hat{C}$ and the corresponding mean square errors for the populations of men, women and all the COPD patients.

$$\begin{array}{c} \begin{array}{cc} \text{Men}: & \\ \hat{C}={\left[0.4877,\;1.544,\;2.106,\;4.194,\;2.50,\;4.429,\;5.879\right]}^{T}, & RMSE=0.5108\\ \text{Women}: & \\ \hat{C}={\left[0.4866,\;1.415,\;1.99,\;4.494,\;2.68,\;3.945,\;5.798\right]}^{T}, & RMSE=0.5396\\ \text{All}\;\text{patients}: & \\ \hat{C}={\left[0.494,\;1.486,\;2.081,\;4.099,\;2.602,\;4.255,\;5.814\right]}^{T}, & RMSE=0.5259 \end{array} \end{array}$$

Unsurprisingly, RMSE values while using models (8) and (9) are quite close to each other. We can preliminary say that both models can be (almost) equally accurate for the modeling task considered.

### Cross-validation for model selection

**Fig 7:**
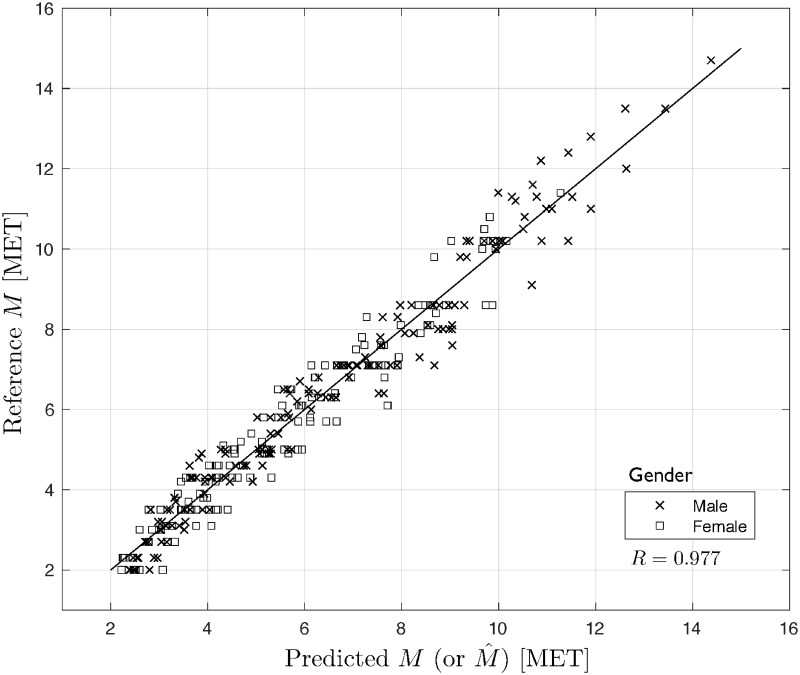
Reference vs. predicted values of *M* for polynomial model.

**Fig 8:**
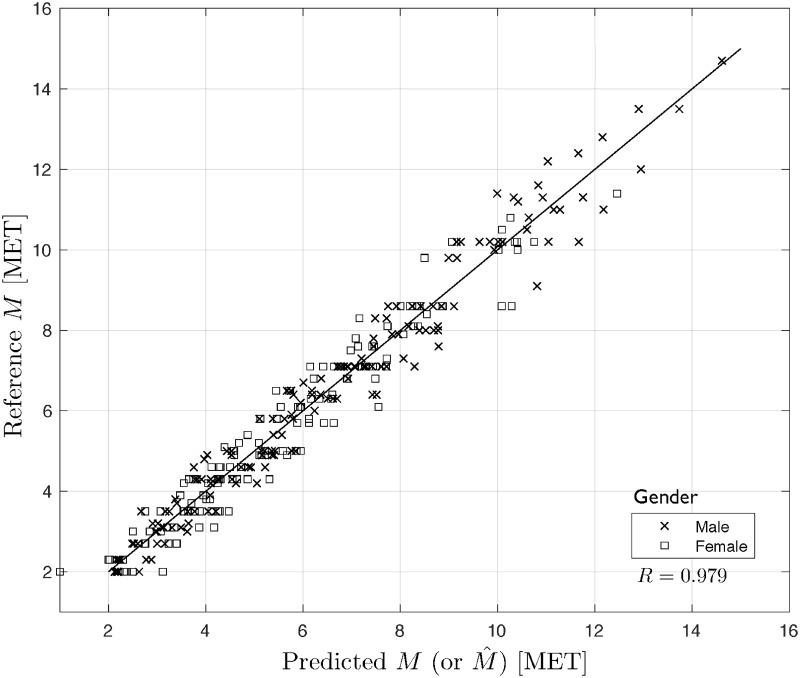
Reference vs. predicted values of *M* for piecewise linear model.

It is well known that an LS fit to data can be assessed with various model selection criteria, see e.g. https://en.wikipedia.org/wiki/Model_selection. In our case, we choose a popular model validation technology which is cross-validation (CV) [53, 54]], in particular Leave-One-Out (LOO) CV regarding its VO2max-related modeling applications [5, 55]. LOO CV uses (*N* − 1) input/output data points for training and the remaining single input/output data point for validation, that is a prediction is made for that point. This is repeated *N* times for all the training/validation sets. The *RMSE*_*cv*_ error for LOO CV, over all those validation points, is computed and used to evaluate the model. We have developed a Matlab-scripted program to LOO cross-validate our two classes of models. The obtained results for the men, women and all patients populations, respectively, are presented in Table 2 and Figs 7 and 8.

**Table 2:** Results of LOO CV for polynomial and piecewise linear models.

Model	Results
	**Men**
polynomial model	*RMSE*_*cv*_ = 0.5779
piecewise linear model	*RMSE*_*cv*_ = 0.5429
	**Women**
polynomial model	*RMSE*_*cv*_ = 0.5401
piecewise linear model	*RMSE*_*cv*_ = 0.5993
	**All patients**
polynomial model	*RMSE*_*cv*_ = 0.5666
piecewise linear model	*RMSE*_*cv*_ = 0.5608

As seen from Table 2, the values of the performance measures are quite close to those *RMSE*s obtained above in this Section. Also, Figs 7 and 8 present a very nice prediction quality of the considered models, which translates to very good properties of the residuals (not illustrated here), compare [5, 53–55]. Additionally, the Pearson correlation coefficients *R* are very high for both cases. This confirms the validity of the presented polynomial expansion and piecewise linear models.

## Discussion

Clearly, the linear model (6) or (7) is unacceptable, in general. The performances of the two nonlinear models considered are comparable for the study populations under tests. Even though the parameter estimation problem is highly nonlinear for the piecewise linear model (9), which necessitates the employment of rather complicated, genetic minimization algorithms, we still opt for using that model. The reason is our rehabilitation application of the model. In our specific case, COPD patients were subjected to both treadmill and 6MWT tests in order to formulate relationships between results of the two tests. Now, having constructed the models (9) we would be able to use a result of the simple 6MWT, instead of the treadmill exercise test, to assign the patient to one of four rehabilitation groups. Why four? No one precisely knows but the rehabilitation practices, both cardiac and pulmonary ones, have developed some routines subdividing the sick population into four rehabilitation groups or classes. That is why our model (9) comprises *n* = 4 linear segments. Why linear and not e.g. cubic splines? Just for simplicity. Note that we optimize the locations of change points *V*_*i*_s (in addition to parameter estimates) and this corresponds with ‘optimization’ of their MET switch points $M^{\left(i\right)}={\hat{M}}_{pl}\left(V_{i}\right)$, *i* = 1, …, *n*, that can be used in the patients’ division into rehabilitation classes. And yet, final selections of *M*^(*i*)^s in the rehabilitation procedures may be not unique, they may change from country to country or even from hospital to hospital. For example, let us recall a (rather intuitive) division into cardiopulmonary rehabilitation groups typically encountered in the therapeutic society, also in Poland:

$$\begin{array}{c} \begin{array}{cc} \text{Group}\;A(\text{less}\;\text{sick}): & M\geq 7.0\;\text{METs}\\ \text{Group}\;B(\text{less}\;\text{medium}): & 5.0\leq M<7.0\;\text{METs}\\ \text{Group}\;C(\text{more}\;\text{medium}): & 3.0\leq M<5.0\;\text{METs}\\ \text{Group}\;D(\text{severe}\;\text{sick}): & 1.0<M<3.0\;\text{METs} \end{array} \end{array}$$ (11)

Note that the division does not account for gender, the factor apparently affecting the results of exercise tests as can also be seen from our experiments. Let us confront now the above division with the results obtained from the model (9) for our study population. In Figs 4 through 6 we show the optimal locations of the change points for each population case. For lucidity, the ‘optimal’ MET switch points are exemplary depicted only in Fig 5 as *M*^(1)^ = 2.304, *M*^(2)^ = 4.094 and *M*^(3)^ = 7.782. Those are not quite in agreement with the group division presented above, which might suggest some corrections to the classification (11). We use to implement those corrections in our rehabilitation practice indeed, in particular 1) discriminating between two rehabilitation divisions like in (11) with respect to gender and 2) rectifying the boundaries of classes specified in the classification (11).

### Treadmill exercise tests revisited

The crucial problem in treadmill exercise test procedures is estimation of a ‘reference’ nonlinear model relating a final result in walk time *T* [min] (or distance *D* [m] or velocity *v* [km/h]) to its VO2max score or, ‘almost’ equivalently to the (sub)maximal effort tolerance *M* in METs. Abstracting from our 6MWT rehabilitation-oriented application, this is nothing but our model (8) or (9). Now, the problem is that some models sometimes investigated in treadmill exercise test procedures may be far from reality. Let us recall some examples brought from [Wikipedia]. https://en.wikipedia.org/wiki/Bruce_protocol:

$$\begin{array}{l} \text{VO2max}\;\left(\text{ml}/\text{kg}/\text{min}\right)=14.76-\left(1.379\times T\right)+\left(0.451\times T^{2}\right)-\left(0.012\times T^{3}\right)\\ \text{Women}:\text{VO2max}\;\left(\text{ml}/\text{kg}/\text{min}\right)=2.94\times T+3.74\\ \text{Women}:\;\text{VO2max}\;\left(\text{ml}/\text{kg}/\text{min}\right)=4.38\times T-3.9\\ \text{Men}:\;\text{VO2max}\;\left(\text{ml}/\text{kg}/\text{min}\right)=2.94\times T+7.65\\ \text{Young}\;\text{Men}:\;\text{VO2max}\;\left(\text{ml}/\text{kg}/\text{min}\right)=3.62\times T+3.91 \end{array}$$

Apparently, the above linear models are unacceptable as the phenomenon is nonlinear, in general. Well, get the first model which is nonlinear. However, its offset term is 14.76, which is a disaster because for *T* = 0, which is the resting condition, we should have the oxygen uptake of some 3.5 [ml/kg/min] and this is the value the offset term should be approximately equal to. But for the two linear models we have the offset terms close to 3.5. This means that the two models were constructed for populations of 1) severe sick (women) patients mainly and 2) young men of low effort tolerance mainly. This is in contrast to the linear model with the offset term equal to −3.9, which suggests that the population of low sick or low medium (women) patients was mainly investigated.

Now, treadmill exercise test procedures are equipped with specific, typically nonlinear models but still they may not be population-related. The treadmill exercise test procedures need banks of nonlinear reference models designed for various patient populations and called e.g. from keyboard for each individual patient under test, depending on various conditions including gender, type/class of sickness, possibly age range and others. This might be involving and time-consuming in general and that is why we have sought for the simple 6MWT substitute herein.

Still, we believe the concept of developing the banks of nonlinear models for treadmill exercise test procedures could be attractive sooner or later. The intimation of such an idea is the second goal of this paper. To this end, we can make generally available (upon request) all our programming packages necessary for construction and simulation of models (8) and (9). The final goal would be to develop an expert system for estimation of (sub)maximal effort tolerance for a plethora of patient populations involved in various patient management tasks, not only the rehabilitation one.

**Remark 2**. Similar banks of nonlinear models for treadmill exercise test procedures could be developed for athlete management software, in particular for junior athletes in their preliminary selection process.

**Remark 3**. Even though many VO2max or MET-involving models are single input, with the input variable being either walk time or velocity or distance, the time has come to thoroughly introduce the second input variable into the treadmill exercise test model, that is the grade. Such a more complex and general model is out of scope of this study and it will be a subject of our future research.

### 6MWT revisited

Apart from the above-mentioned, general need for treadmill exercise test procedures to incorporate banks of ‘reference’ nonlinear models (rather than one ‘average’ model), right the same holds for the 6MWT score modeled by Eqs (8) or (9). Our results in METs vs. 6MWT distance/velocity presented hereinbefore could be a starting step for the development of such a bank for COPD patient populations within the mBruce procedure. Again, our software is open not only to the rehabilitation society.

**Remark 4**. Note that e.g. a 6MWT bank for COPD patient populations should strictly ‘cooperate’ with the corresponding COPD bank within the mBruce procedure. Unfortunately, this ‘compatibility rule’ is currently rather seldom observed during treadmill exercise tests, that is e.g. COPD patients are tested against some nonlinear reference model of VO2max vs. *T*, being likely a sort of an ‘average’ characteristic for cardiopulmonary patients.

## Conclusions

Having backgrounded the energy expenditure process due to physical activity, we justified the nonlinear relationship between VO2max and power output during a submaximal exercise test. This has led to the proposal of two classes of nonlinear models, namely polynomial expansion and piecewise linear models. The models can be used to estimate submaximal effort tolerance in patients with COPD by means of the cost-saving 6MWT, without the use of cardiorespiratory analysis. The models’ LS fit to data was successfully assessed by means of the LOO cross-validation technique. The above constitutes an original modeling methodology for estimation of submaximal effort tolerance, which is the main achievement of the paper. Our new nonlinear estimation algorithms have been finally applied for piecewise linear models in rehabilitation of COPD patients, improving their assignment rules to four rehabilitation classes. The second achievement of the paper has been the proposal to use our nonlinear estimation methodology in treadmill exercise test procedures in order to construct a bank of (nonlinear) ‘reference’ models cooperating compatibly with various patient populations under various patient management environments. This challenging, expert system-oriented future research direction will be followed in our works. Another interesting future research topic could be an extension of our methodology to self-paced treadmill exercise tests, combined with 6MWT, compare [37]. Yet another challenging research topic is incorporation of the second input variable, that is treadmill grade, into our nonlinear single-input models relating energy expenditure in METs with either 6MWT or treadmill test scores.

## Supporting information

S1 Table Dataset related with 6MWT for men’s population. (TXT)

S2 Table Dataset related with 6MWT for women’s population. (TXT)
